# KBG syndrome: videoconferencing and use of artificial intelligence driven facial phenotyping in 25 new patients

**DOI:** 10.1038/s41431-022-01171-1

**Published:** 2022-08-15

**Authors:** Lily Guo, Jiyeon Park, Edward Yi, Elaine Marchi, Tzung-Chien Hsieh, Yana Kibalnyk, Yolanda Moreno-Sáez, Saskia Biskup, Oliver Puk, Carmela Beger, Quan Li, Kai Wang, Anastassia Voronova, Peter M. Krawitz, Gholson J. Lyon

**Affiliations:** 1 Department of Human Genetics, NYS Institute for Basic Research in Developmental Disabilities, 1050 Forest Hill Road, Staten Island, NY 10314 USA; 2 Institute for Genomic Statistics and Bioinformatics, University Hospital Bonn, Rheinische Friedrich-Wilhelms-Universität Bonn, Bonn, Germany; 3 Department of Medical Genetics, Faculty of Medicine & Dentistry, University of Alberta, Edmonton, AB Canada; 4 Department of Cell Biology, Faculty of Medicine & Dentistry, University of Alberta, Edmonton, AB Canada; 5 Medical Genetic Unit, Sistemas Genómicos, Valencia, Spain; 6 CeGaT GmbH, Praxis für Humangenetik Tübingen, Tübingen, Germany; 7 MVZ Labor Krone GbR, Filialpraxis für Humangenetik, Bielefeld, Germany; 8 Princess Margaret Cancer Centre, University Health Network, University of Toronto, Toronto, ON M5G2C1 Canada; 9 Raymond G. Perelman Center for Cellular and Molecular Therapeutics, Children’s Hospital of Philadelphia, Philadelphia, PA 19104 USA; 10 George A. Jervis Clinic, NYS Institute for Basic Research in Developmental Disabilities, 1050 Forest Hill Road, Staten Island, NY 10314 USA; 11 Biology PhD Program, The Graduate Center, The City University of New York, New York, NY USA

**Keywords:** Genetic predisposition to disease, Genetics research

## Abstract

Genetic variants in Ankyrin Repeat Domain 11 (*ANKRD11*) and deletions in 16q24.3 are known to cause KBG syndrome, a rare syndrome associated with craniofacial, intellectual, and neurobehavioral anomalies. We report 25 unpublished individuals from 22 families with molecularly confirmed diagnoses. Twelve individuals have *de novo* variants, three have inherited variants, and one is inherited from a parent with low-level mosaicism. The mode of inheritance was unknown for nine individuals. Twenty are truncating variants, and the remaining five are missense (three of which are found in one family). We present a protocol emphasizing the use of videoconference and artificial intelligence (AI) in collecting and analyzing data for this rare syndrome. A single clinician interviewed 25 individuals throughout eight countries. Participants’ medical records were reviewed, and data was uploaded to the Human Disease Gene website using Human Phenotype Ontology (HPO) terms. Photos of the participants were analyzed by the GestaltMatcher and DeepGestalt, Face2Gene platform (FDNA Inc, USA) algorithms. Within our cohort, common traits included short stature, macrodontia, anteverted nares, wide nasal bridge, wide nasal base, thick eyebrows, synophrys and hypertelorism. Behavioral issues and global developmental delays were widely present. Neurologic abnormalities including seizures and/or EEG abnormalities were common (44%), suggesting that early detection and seizure prophylaxis could be an important point of intervention. Almost a quarter (24%) were diagnosed with attention deficit hyperactivity disorder and 28% were diagnosed with autism spectrum disorder. Based on the data, we provide a set of recommendations regarding diagnostic and treatment approaches for KBG syndrome.

## Introduction

KBG syndrome (OMIM:148050), first described by Herrmann et al. [1], is named after the surnames (K-B-G) of the first families reported with the syndrome. The original report described anomalies such as short stature, skeletal abnormalities, cognitive disability, and specific craniofacial dysmorphisms. Subsequent research has expanded the list of anomalies to include seizures, behavioral disturbances, congenital heart defects, and gastrointestinal issues [2–8**]**. Genetic variants in Ankyrin Repeat Domain 11 (*ANKRD11*) and deletions in 16q24.3 are known to cause KBG syndrome [9]. One author (GJL) was introduced to KBG syndrome by an original describer of the syndrome (John Opitz), and published a case report describing a 13-year-old boy with epilepsy, severe developmental delay, distinct facial features, and hand anomalies [10]. The very serious nature of his epilepsy and its subsequent negative impact on development was notable. In the present study, GJL met and interviewed 25 individuals with KBG syndrome to better characterize the disease and investigate the effects of epilepsy and other conditions on the trajectory of neurodevelopment in individuals with KBG syndrome. Additionally, a facial photograph could ideally be combined with medical records and variant prioritization efforts, after exome or genome sequencing, to accurately classify new pathogenic missense and other variants in rare syndromes. We assess the current state of two leading facial recognition software algorithms [11, 12] and demonstrate the use of a variant prioritization approach, PEDIA, [13] that integrates phenotypic features, facial images, and exome data.

## Methods

Twenty-five individuals (11 females, 14 males) from 22 families throughout eight countries were interviewed via Zoom (version 5.2.0) by a single physician (GJL) over a 4-month period from February 2021 to June 2021. All interviews were conducted in English, with a translator used for one family whose primary language was Spanish. Interviews were approximately one to two hours long and consisted of structured questions and the physician’s visual assessment of facial and limb phenotypic characteristics.

All patients interviewed were molecularly diagnosed with KBG syndrome and were self-referred or recruited via a private Facebook group created by the KBG Foundation. Genetic reports, medical records including imaging, and photos (facial and whole-body) were collected from families by email and compiled prior to the interviews. Photo consent was obtained. The height and weight at the time of videoconference was obtained via verbal report or documented from the most recent medical reports and growth charts.

All variants were annotated to the NM_013275.5 transcript in GrCh37/hg19. Every reported anomaly was documented as a standardized Human Phenotype Ontology (HPO) term and compiled on the open-source Human Disease Genes (HDG) website series to promote international data sharing [14]. The presence of a trait or phenotype was documented when it was explicitly stated in the interview or found in the individual’s medical records. Facial photos provided by the families and/or taken by the clinician during videoconferencing were loaded onto Face2Gene (version 20.1.4; FDNA Inc, USA) [11] and GestaltMatcher (version 1.0) through Bonn University [12]. DeepGestalt photos and phenotype data were uploaded on August 24, 2021 (at which time the GestaltMatcher algorithm was not available in Face2Gene). These programs use deep convolutional neural networks to build syndrome and patient classifiers, respectively.

**Fig. 1:**
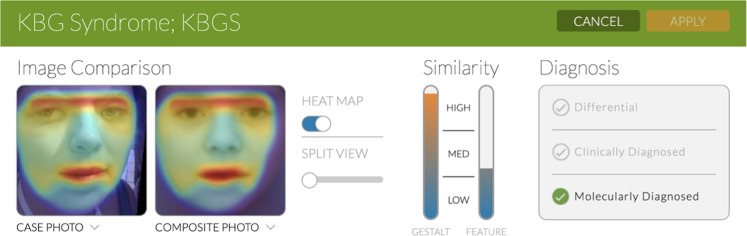
Illustration of Face2Gene. DeepGestalt results of Individual R indicating high gestalt and medium feature scores. Heatmap visualization shows goodness-of-fit between areas of the individual’s image and the suggested syndrome, KBG.

Face2Gene (F2G) uses several different algorithms, including DeepGestalt, a facial phenotyping framework that measures the similarity between a patient and a specific genetic disorder. Its algorithm is trained using images of individuals from many different genetic syndromes. Once a photo is uploaded, the software provides a ‘gestalt score’, with a higher score indicating greater similarities in facial morphology to a specific disorder [11]. In addition to a photo, the physician can input relevant phenotypic features (e.g., anteverted nares, prominent nasal bridge) which are used to derive a ‘feature score’, an indicator of how well the clinical text seems to fit a specific diagnosis. The gestalt and feature scores, ranked high, medium, or low, are further combined to produce a list of the top 30 syndromes that the individual most closely matches. This “combined score” is based on an optimization of a test set proprietary to FDNA. The clinician then confirms the diagnosis as a “differential”, “clinically diagnosed” or “molecularly diagnosed” (Fig. 1).

GestaltMatcher, an extension of DeepGestalt, quantifies the similarity in features between two patients with KBG, allowing for the identification of syndrome-specific genetic traits. Unlike DeepGestalt which quantifies similarities on a syndromic level, GestsaltMatcher quantifies similarities between images and returns a score of similarity for various individuals with one specified syndrome. We uploaded pictures of the 25 individuals with KBG syndrome and calculated the degree of overlap in facial features.

Furthermore, we used a variant prioritization approach, PEDIA (version 1.1) [13], which uses a facial photo, clinical features (HPO terms) and exome sequencing data as input. PEDIA integrates the score of each gene calculated from DeepGestalt, Case Annotations, as well as Disease Annotations (CADA) [15] and Combined Annotation–Dependent Depletion (CADD) [16]. DeepGestalt first derives the gestalt score of each gene, then a CADA score of each gene is calculated (https://cada.gene-talk.de/webservice/) for feature analysis. Since only the disease-causing variant of each patient was known, we performed the exome simulation by inserting the disease-causing variants into a randomly selected exome from the 1000 Genomes Project [17] to obtain the genomic score. We then annotated the exome variants with a CADD score [16], using the highest CADD score among the variants of each gene. The PEDIA model was trained with the gestalt, CADA, and CADD scores and used to calculate likelihood scores for each gene for all 25 patients. These scores were sorted in descending order, with the top-1, top-10 and top-30 accuracy reported.

The annotation of variants in ClinVar for Supplementary Table 2 is described in further detail in Supplementary Methods.

## Results

### Molecular findings

**Fig. 2:**
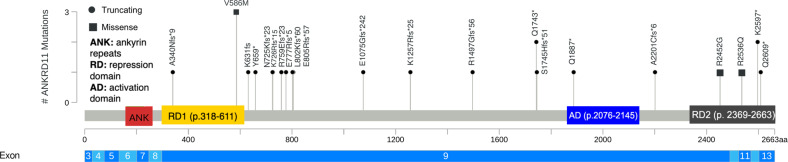
Schematic representation of DNA and protein variants of the 22 families along *ANKRD11*. The coding exons for *ANKRD11* are depicted to scale. Abbreviations: aa amino acid. The figure was made using: https://www.cbioportal.org/mutation_mapper.

The variants occurred de novo in 12 individuals, were maternally inherited in Individuals K and L, and paternally inherited in individual O. One parent of affected Individual T, Individual U, showed a low level of mosaicism for the variant (with only 2 out of 298 sequencing reads for this variant found in her blood). Nine individuals had unknown modes of inheritance. A majority, 20, are truncating variants (frameshift or nonsense), and five are missense (with three of five belonging to the same family). Twenty-one distinct variants were identified (Table 1), with locations shown in Fig. 2 [18].

**Table 1:** Cohort characteristics.

Individual	Gender	Age at time of videoconference (years)	g.DNA (Hg19/GRCh37)	cDNA (NM_013275.6)	Protein effect	Variant type	Mode of Inheritance
A	Male	21–25	NC_000016.9:g.89351932dup	c.1018dup	p.(Thr340Asnfs*9)	Frameshift	Unknown
B	Female	6–10	NC_000016.9:g.89350619_89350622del	c.2329_2332del	p.(Glu777Argfs*5)	Frameshift	Unknown
C	Male	6–10	NC_000016.9:g.89345596G>C	c.7354C>G	p.(Arg2452Gly)	Missense	De novo
D	Female	31–35	NC_000016.9:g.89347717_89347718del	c.5233_5234del	p.(Ser1745Hisfs*51)	Frameshift	Unknown
E	Female	5–10	NC_000016.9:g.89350544_89350547del	c.2404_2407del	p.(Leu802Lysfs*60)	Frameshift	De novo
F	Female	21–25	NC_000016.9:g.89350773_89350774del	c.2177_2178del	p.(Lys726Argfs*15)	Frameshift	De novo
G	Male	5–10	NC_000016.9:g.89348460_89348461del	c.4489_4490del	p.(Arg1497Glyfs*56)	Frameshift	De novo
H	Male	11–15	NC_000016.9:g.89335053G>A	c.7825C>T	p.(Gln2609*)	Nonsense	de novo
I	Male	5–10	NC_000016.9:g.89341328C>T	c.7607G>A	p.(Arg2536Gln)	Missense	De novo
J	Male	5–10	NC_000016.9:g.89349180_89349181del	c.3770_3771del	p.(Lys1257Argfs*25)	Frameshift	de novo
**K**	**Male**	**5–10**	**NC_000016.9:g.89351194C>T**	**c.1756G>A**	**p.(Val586Met)**	**Missense**	**Maternal**
**L**	**Male**	**0–4**	**NC_000016.9:g.89351194C>T**	**c.1756G>A**	**p.(Val586Met)**	**Missense**	**Maternal**
**M**	**Female**	**36–40**	**NC_000016.9:g.89351194C>T**	**c.1756G>A**	**p.(Val586Met)**	**Missense**	**Unknown**
N	Male	0–4	NC_000016.9:g.89350777_89350780del	c.2175_2178del	p.(Asn725Lysfs*23)	Frameshift	De novo
**O**	**Male**	**21–25**	**NC_000016.9:g.89337242T>A**	**c.7789A>T**	p.(Lys2597*)	**Nonsense**	**Paternal**
**P**	**Male**	**55–60**	**NC_000016.9:g.89337242T>A**	**c.7789A>T**	p.(Lys2597*)	**Nonsense**	**Unknown**
Q	Female	11–15	NC_000016.9:g.89350677dup	c.2273dup	p.(Arg759Glu*23fs)	frameshift	Unknown
R	Female	20–25	NC_000016.9:g.89346353_89346354insT	c.6596_6597insA	p.(Ala2201Cysfs*6)	Frameshift	De novo
S	Female	11–15	NC_000016.9:g.89347291G>A	c.5659C>T	p.(Gln1887*)	Nonsense	Unknown
**T**	**Male**	**5–10**	**NC_000016.9:g.89347723G>A**	**c.5227C>T**	p.(Gln1743*)	**Nonsense**	**Maternal**
**U**	**Female**	**30–35**	**NC_000016.9:g.89347723G>A**	**c.5227C>T**	p.(Gln1743*)	**Nonsense**	**Unknown**
V	Female	5–10	NC_000016.9:g.89350973G>C	c.1977C>G	p.(Tyr659*)	Nonsense	de novo
W	Male	5–10	NC_000016.9:g.89350540_89350543del	c.2409_2412del	p.(Glu805Argfs*57)	Frameshift	De Novo
X	Female	16–20	NC_000016.9:g.89351062del	c.1893del	p.(Lys631fs)	Frameshift	De Novo
Y	Male	16–20	NC_000016.9:g.89349727_89349730del	c.3224_3227del	p.(Glu1075Glyfs*242)	Frameshift	Unknown

This cohort includes 14 males and 11 females ranging from ages 1 year to 59 years.

Truncating variants are classified by ACMG criteria [19] as: “PVS1 null variant (nonsense, frameshift) in a gene where loss of function is a known mechanism of disease.” Some variants are classified as “PS2 *De novo* (both maternity and paternity confirmed) in a patient with the disease and no family history”. One missense variants in our cohort (p. (Val586Met) was seen in a heterozygous control individual in the Genome Aggregation Database (GnomAD), thus calling into question its pathogenicity. It is also formally possible that the one individual in GnomAD might be mildly affected. The mother with this variant (individual M) has a very mild phenotype whereas her children (individuals K and L) have phenotypes more consistent with KBG syndrome. However, a recent preprint [20] demonstrated that some missense variants do impair ANKRD11 ability and/or stability, but that these variants mainly localize in the Repression Domain 2. Those authors also tested one variant in the Repression domain 1 (p.Leu509Pro), which turned out to have no effect on ANKRD11 stability or activity. The p.(Val586Met) variant of individuals K, L, and M also falls within the Repression Domain 1, and it has a borderline CADD score (23.9) and is not as highly conserved as the other missense variants. In addition, the affected nucleotides and corresponding amino acid are also not highly conserved when the sequence is aligned with other species. Per DeepGestalt, these individuals (K, L, M) did not have KBG syndrome listed in their top 30 differentials. Segregation analysis with the mother and sister of Individual M is not yet available. While the mother has very mild clinical features of KBG syndrome, the sister (aunt of Individuals K and L) is potentially reporting more severe symptoms. Ultimately, the pathogenicity of the variant (p.(Val586Met)) is still uncertain.

A different missense variant (p. Arg2536Gln) arose *de novo* and was initially classified as a variant of uncertain significance because it had not been previously reported. However, it has been reclassified because of new information available: two additional patients carrying the variant. One is reported in Clinvar (https://www.ncbi.nlm.nih.gov/clinvar/variation/1012410/?new_evidence=false), a patient in whom the variant was maternally inherited (referred to as Individual Z in Supplementary Information), but who was unavailable for videoconferencing. In the other previously reported patient, the variant has arisen *de novo* and was classified as pathogenic [21]. Although a more extensive cosegregation of the patient reported in Clinvar is not available, since phenotypes characteristic of KBG syndrome are seen in three individuals possessing this variant, the variant is reclassified to likely pathogenic. Further details about these cases can be found in Supplemental Text and Case Summaries.

As of April 2022, there are 429 putative missense or non-frameshift deletion, substitution or insertion variants in *ANKRD11* submitted to ClinVar [22], with many of these listed as variants of uncertain significance (Supplementary Table 2), with bioinformatic analyses providing a suggested consensus classification for each variant.

Median age of the 25 individuals was 11 years and average age was 15 years (range = 1–59). One comes from a consanguineous family, roughly half (*n* = 12) had a history of congenital abnormalities in the family, and eight had relatives with intellectual disabilities.

The parents of individuals B, D, T, and Y had histories of miscarriage. The variant was *de novo* for individual B, whereas the parent of individual T (Individual U) was mosaic for the missense variant (as noted above). The mother of individual Z has a history of several miscarriages early in pregnancy around six weeks of age. The inheritance pattern is unknown for individuals D and Y.

The parents in this study (M, P, U) generally had mild phenotypic features. Individual M, the mother of K and L, possessed some distinct facial traits (e.g., thick eyebrows, anteverted nares, broad nasal base), however, the overall constellation of features was not typical of KBG syndrome. She did not present with common features such as developmental delay, macrodontia, or short stature. Conversely, individual P, the father of O, presented with global developmental delay, macrodontia, and short stature among other common traits of KBG syndrome. Lastly, individual U, the mother of T, had mild facial features (e.g., synophrys, thick eyebrow, wide nasal bridge, prominent nasal tip) with speech delays and seizures in childhood.

The overall frequency of certain phenotypic features is shown in Table 2, and these are reviewed in further detail in the following sections.

**Table 2:** Frequency of phenotypic abnormalities in 25 KBG video-conferenced participants.

Distinct facial features	
Eyes	
Thick/ long eyebrows	56% (14/25)
Synophrys	44% (11/25)
Long/Prominent eyelashes	32% (8/25)
Hypertelorism	28% (7/25)
Strabismus	28% (7/25)
Nose	
Wide nasal bridge/nose	64% (16/25)
Prominent/broad nasal tip	60% (15/25)
Anteverted nares	52% (13/25)
Abnormal/wide nasal base	32% (8/25)
Mouth	
Macrodontia	64% (16/25)
Thin upper lip vermillion	32% (8/25)
Persistence of primary teeth	16% (4/25)
Tented philtrum	12% (3/25)
Ears	
Low-set ears	16% (4/25)
Large/prominent ears	8% (2/25)
Posteriorly rotated ears	4% (1/25)
Attached lobe	4% (1/25)
Anteverted ears	4% (1/25)
Intellectual disability	72% (18/25)
None	8% (2/25)
Mild	56% (14/25)
Moderate	12% (3/25)
Severe	4% (1/25)
Behavioral abnormalities	84% (21/25)
Abnormal mood	48% (12/25)
Aggressive violent behavior	28% (7/25)
Autism Spectrum Disorder	28% (7/25)
Self-injurious behavior	20% (5/25)
Impulsivity	16% (4/25)
Repetitive compulsive behavior	8% (2/25)
Cardiac abnormalities	52% (13/25)
Patent foramen ovale	12% (3/25)
Ventricular septal defect	12% (3/25)
Persistent left superior vena cava	8% (2/25)
Atrial septal defect	8% (2/25)
Heart murmur	8% (2/25)
Abnormal mitral valve morphology	8% (2/25)
Tetralogy of Fallot	4% (1/25)
Atrial fibrillation	4% (1/25)
Patent ductus arteriosus	4% (1/25)
Neurological abnormalities	96% (24/25)
Motor delay	88% (22/25)
Developmental delay	80% (20/25)
Speech delay	72% (18/25)
Hypotonia	56% (14/25)
Language delay	52% (13/25)
Seizures	44% (11/25)
Gait disturbance	40% (10/25)
Autism spectrum disorder	28% (7/25)
Migraines	24% (6/25)
ADHD	24% (6/25)
Skeletal abnormalities	100% (25/25)
Short stature	60% (15/25)
Clinodactyly	48% (12/25)
Clinodactyly of the 5th digit	50% (6/12)
Pes Planus	40% (10/25)
Sacral dimples	28% (7/25)
Scoliosis (thoracic, lumbar, kyphoscoliosis)	24% (6/25)
Osteoporosis/ Osteopenia	20% (5/25)
Delayed fontanel closure	20% (5/25)
Small hands	16% (4/25)
Brachydactyly	16% (4/25)
Kyphosis	16% (4/25)
Abnormal or single palmar crease	12% (3/25)
Abnormality of the ankle/Ankle clonus	12% (3/25)
Abnormality of head shape	16% (4/25)
Macrocephaly	8% (2/25)
Microcephaly & Dolichocephaly	4% (1/25)
Brachycephaly	4% (1/25)
Gastrointestinal abnormalities	80% (20/25)
GERD	44% (11/25)
Feeding difficulties in infancy	44% (11/25)
Vomiting	36% (9/25)
Chronic constipation	28% (7/25)
NG/G tube feeding	20% (5/25)
Abdominal pain/ migraines	20% (5/25)
Hiatal/Inguinal hernia	12% (3/25)
Ears, nose, throat and vision	
Vision Impairment	60% (15/25)
Astigmatism	24% (6/25)
Myopia	24% (6/25)
Hyperopia	20% (5/25)
Strabismus	16% (4/25)
Nystagmus	8% (2/25)
Hearing loss	44% (11/25)
Conductive	20% (5/25)
Unspecified	16% (4/25)
Sensorineural	4% (1/25)
Mixed	4% (1/25)
Palate abnormalities (soft, high, narrow)	24% (6/25)
Ankyloglossia	12% (3/25)
Retrognathia	8% (2/25)
Endocrine, immune, metabolic	
Precocious puberty	8% (2/25)
Thyroid Abnormality	8% (2/25)
Immune System Abnormality	
Allergies	32% (8/25)
Rheumatoid arthritis	8% (2/25)
Metabolic abnormality	
Slender build	20% (5/25)
Failure to thrive	24% (6/25)

### Stature

Height at the time of videoconference clustered into 3–98th centile (44%), below 3rd centile (24%) and above 98th centile (12%) with a median height of 140.0 ± 29.4 cm. Weights at time of videoconference clustered into 3-98th centile (48%), below the 3rd centile (20%), and above 98th centile (4%), with a median weight of 27.8 ± 29.1 kg. Of the three individuals who had heights above the 98th centile at time of videoconference, one had been put on growth hormone for approximately 2–4 years (Individual J) (Table 3). Birth length clustered into 3–98th centile (44%), above 98th centile (8%), and below 3rd centile (8%), with a median length of 49.0 ± 6.3 cm. Birth weight clustered between 3–98th centile (64%), and below the 3rd centile (16%) with a median birth weight of 3 ± 0.7 kg.

**Table 3:** Height and weight centiles for KBG participants at time of videoconference and at birth.

Individual	Current height (cm)	Height percentile (SD)	Current weight (kg)	Weight percentile (SD)	Birth length (cm)	Length percentile (SD)	Birth weight (kg)	Birth weight percentile (SD)
A	163	3 (−1.89)	68	45 (−0.13)	“Normal”	Unknown	3	18 (−0.90)
B	118	73 (+0.61)	23	73 (+0.61)	49	42 (−0.20)	3.27	38 (−0.30)
C	127	4 (−1.80)	23.5	2 (−2.02)	Unknown	99	Unknown	Unknown
D	150	2 (−2.04)	Unknown	Unknown	Unknown	Unknown	Unknown	Unknown
E	99	3 (−1.89)	18.2	53 (+0.08)	50.8	69 (+0.49)	3.46	53 (+0.08)
F	140	<1 (−3.58)	32	<1 (−3.80)	31	<1 (−8.28)	0.773	<1 (−5.05)
G	113.4	<1 (−3.91)	22.7	1 (−2.22)	Unknown	Unknown	2	1 (−2.50)
H	Unknown	Unknown	Unknown	unknown	Unknown	Unknown	3.3	34 (−0.42)
I	105	> 99 (+3.47)	16	93 (+1.49)	46	6 (−1.56)	2.73	9 (−1.33)
J	115.6	13	21.2	24	43.18	<1 (−2.61)	3.65	57 (+0.18)
**K**	**Unknown**	**Unknown**	**Unknown**	**Unknown**	**56**	**99 (+2.23)**	**3.27**	**32 (−0.47)**
**L**	**Unknown**	**95**	**11.8**	**24 (−0.71)**	**Unknown**	**Unknown**	**Unknown**	**Unknown**
**M**	**177.8**	**99 (+2.25)**	**“Normal”**	**Unknown**	**Unknown**	**Unknown**	**Unknown**	**Unknown**
N	79.4	8 (−1.40)	9.4	1 (−2.26)	50.8	60 (+0.24)	3.23	30 (−0.53)
**O**	**157.48**	**<1 (−2.65)**	**68.03**	**45 (−0.12)**	**47**	**12 (−1.18)**	**2.8**	**11 (−1.22)**
**P**	**157.48**	**<1 (−2.66)**	**58.97**	**11 (−1.22)**	**Unknown**	**Unknown**	**Unknown**	**Unknown**
Q	Unknown	Unknown	Unknown	Unknown	Unknown	Unknown	3.26	38 (−0.31)
R	162.6	46 (−0.10)	58	52 (+0.04)	46.5	9 (−1.32)	2.4	3 (−1.95)
S	129.54	2 (−2.02)	37.6	52 (+0.04)	unknown	unknown	3.03	23 (−0.75)
**T**	**Unknown**	**Unknown**	**Unknown**	**Unknown**	**49.5**	**40 (−0.25)**	**3.4**	**40 (−0.26)**
**U**	**Unknown**	**Unknown**	**Unknown**	**Unknown**	**Unknown**	**Unknown**	**1.9**	**<1**
V	Unknown	40–50	unknown	40–50	49.53	51(+0.03)	2.948	18 (−0.91)
W	117	5 (−1.65)	18.9	2 (−2.10)	Unknown	9	3	18 (−0.90)
X	160.02	34 (−0.40)	66.22	76 (+0.70)	49	7 (−1.50)	2.18	1 (−2.37)
Y	182	92 (+1.43)	115	>99 (+3.80)	46.99	12 (−1.19)	2.72	9 (−1.35)

### Facial features

**Fig. 3:**
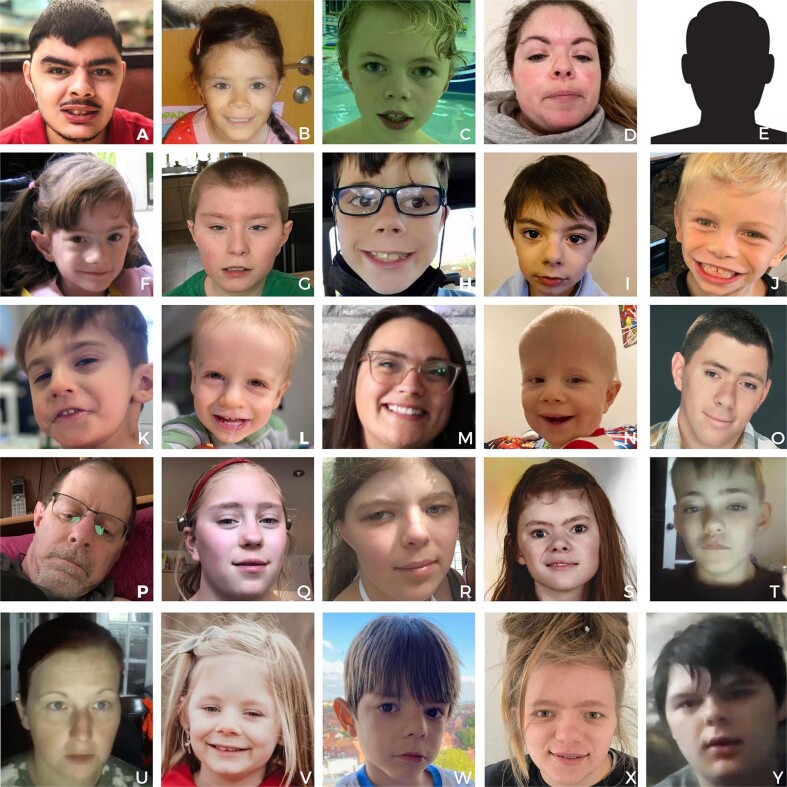
Clinical features of KBG syndrome. Characteristic features include bushy eyebrows (A, C, D, E, I, K, M, O, P, R, T, U, V, Y), long eyelashes (C, D, I, L, O, P, S, X,), triangular face (A, G, K, R, V) and most had a wide nasal bridge or tip and a thin upper vermillion.

The photographs with permission for publication are shown in Fig. 3. At least one distinctive facial feature common to KBG patients was present in every individual interviewed. Defining facial characteristics include thick eyebrows with synophrys, prominent eyelashes, wide nose, thin upper lip vermillion, and macrodontia. Many have a triangular face or pointed chin and a broad or prominent forehead.

#### GestaltMatcher results

**Fig. 4:**
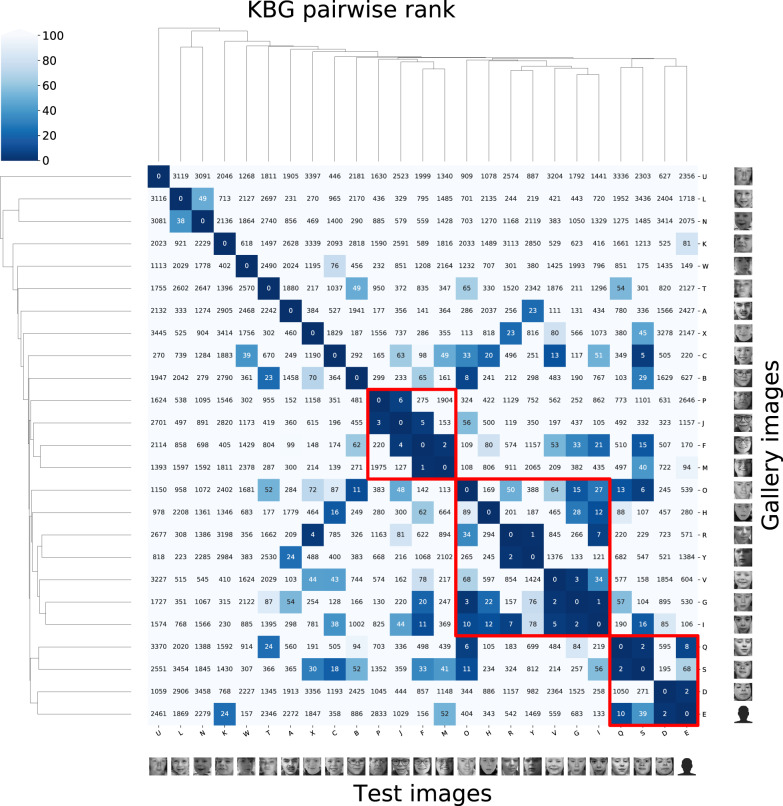
GestaltMatcher results. Sub-cluster P, J, F, M present with synophrys and wide noses. Sub-cluster O, H, R, Y, V, G, I present with thick eyebrows, prominent/broad nasal tips, macrodontia, triangular faces and pointed chins. Sub-cluster Q, S, D, E present with anteverted nares, broad nasal tips, and macrodontia. Link: https://db.gestaltmatcher.org/; individual links to each patient in Supplemental Text. Note: Individual E did not consent to having their photo published, however, a frontal photo was input into the GestaltMatcher and DeepGestalt algorithms.

Pairwise ranks of the 25 photos in Fig. 4 suggest most patients described in this analysis share similar facial phenotypes. In a gallery of 3533 images with 816 different disorders and 25 KBG patients, 15 out of 25 KBG patients had at least one other KBG patient in their top-10 rank, and 21 out of 25 patients had at least one other patient in their top-30 rank. Other than U being an outlier, there was a cluster containing the set of patients with three sub-clusters (P, J, F, and M), (O, H, R, Y, V, G, and I), and (Q, S, D, and E). Patient U was an outlier, perhaps due to the low-level mosaicism for this variant. No clear clusters were seen when segregated by type of genetic variant (missense, frameshift, nonsense). The similarity between family members is a known confounder in the analysis of syndromic similarity. On average, family members with the same disorder are closer in the clinical face phenotype space than unrelated individuals with the same disorder. That said, in one family, we do not see an increased similarity between M, K, and L.

#### DeepGestalt results

KBG syndrome was recommended among the top 30 syndromes and ranked as the first (i.e., most likely) diagnosis for 28% (*n* = 7) of individuals, second for 40% (*n* = 10), and third or fourth for 12% (*n* = 3). Overall, 80% (*n* = 20) of patient’s photos analyzed had KBG syndrome ranked in their top-five potential diagnoses out of the 30 possible suggested syndromes from among the 300+ syndromes currently recognized by the DeepGestalt algorithm. Among the 20 with KBG in the top-five rank, seven had a high gestalt score, 10 had medium gestalt, and three had low gestalt. Fourteen had a medium feature score, five had a low score, and one was unranked for features of KBG (see Supplementary Table 3). Individuals B, F, and J initially submitted photos where they were wearing glasses. After analyzing photos without glasses, the ranking of KBG surprisingly dropped from two to six for individual B and from two to three for individual J. Ranking did not change for individual F. While KBG ranking fluctuated, the gestalt and feature levels did not change between the photos with and without glasses for any of the three individuals.

Five individuals (K, L, M, P, U) did not have KBG syndrome appear as a differential diagnosis out of 30. First ranked diagnoses instead included Cornelia de Lange, Williams-Beuren, Rubinstein-Taybi, Angelman, and mucopolysaccharidosis. Notably, Individual P was 55–60 years old at the time of the videoconference whereas Individual U was 30–35 years old, and both of them initially submitted pictures of themselves around those ages. These ages fall above our median age of 11 years and the age at which most individuals are diagnosed with KBG syndrome. DeepGestalt relies on the photos that it is trained on, so older age photos may not perform as well. Additionally, individual U has very low-level mosaicism for this variant, potentially resulting in lower phenotypic expression of facial features. The other three individuals who were unranked (K, L, and M) are all from the same family and possess the same missense variant (Table 1) with questionable pathogenicity.

#### Variant prioritization with facial images

With PEDIA score, the disease-causing gene *ANKRD11* is ranked at the first place in 18 out of 25 (top-1 accuracy: 72%). When looking at the top-10 genes, *ANKRD11* is listed in the top-10 genes in 22 out of 25 (top-10 accuracy: 88%). All have *ANKRD11* in their top-30 genes.

#### Cognition and neurologic features

Eight reported an intelligence quotient (IQ) score, with a mean of 73 ± 4.84 (range = 64–80) as measured by the Weschler Intelligence Scale (3rd to 5th edition). A majority, 68% are considered mildly to moderately intellectually disabled based on level of functioning. Global developmental delays prior to 5 years were seen in 68% (*n* = 17), with nine being classified as mild. Median age of crawling onset was 12 months (range = 9–24) (*n* = 8), walking onset 22 months (range = 12.5–36) (*n* = 10), and speech onset 30 months (range = 19–36) (*n* = 6). Selective mutism and absent speech were observed in three individuals.

Common types of seizures reported included myoclonic, tonic-clonic, and absence with no specific type predominating [23]. Electroencephalogram (EEG) abnormalities were documented in three of 11 individuals with seizures. According to maternal report, Individual E was meeting speech and motor milestones until the onset of myoclonic seizures, complex partial seizures, and verbal tonic seizures with respiratory distress around 0.5–2 years of age. Similarly, individuals H, K, R, S, T, U, X, and Y reported histories of various types of seizures and concurrent speech and motor delays. Brain abnormalities detected on magnetic resonance imaging (MRI) included pineal cyst, arachnoid cyst, choroid plexus cyst, subdural hemorrhage, and small pituitary gland.

Abnormal mood included abnormal emotion or affect, depression, and/or anxiety, self-injurious behavior including self-biting. Individuals E, O, Q, and R report absent or high pain threshold. O has a history of a fractured foot and a dislocated kneecap with bone scans showing normal density. Impaired tactile sensation was reported in two individuals (M,S).

#### Ear, nose, throat (ENT) and vision

Six had chronic otitis media, with five of six having concurrent hearing impairment. Those experiencing chronic otitis media likewise had a preauricular pit, abnormal or blocked Eustachian tubes, abnormality of the tympanic membrane, enlarged vestibular aqueduct, choanal atresia, and increased size of nasopharyngeal adenoids. Hearing loss and recurrent infections including sinus, chronic ear, and upper respiratory infections were present in four individuals (O, P, Q, Y). Of the six with palatal anomalies, four had difficulties feeding.

#### Skeletal features

Of note, individual A was diagnosed with osteopenia, and later osteoporosis, at 15–20 years with low bone mineral densitometry in the lumbar spine, hip, and femoral neck. An x-ray of his left hand and wrist was performed which revealed physeal closure of the bones, excluding delayed bone maturation. Individual S has visible sacral dimple and was referred to neurology for gait disturbance and urinary incontinency. MRI of her lumbar spine revealed a tethered spinal cord.

#### Cardiovascular features

Cardiac abnormalities were seen in approximately half the participants and while many resolved without the need for surgical intervention, individual K had Tetralogy of Fallot with pulmonary valve-sparing surgical repair at ~3–6 months of age. Individual T had mitral valve repair at around one year of age.

#### Gastrointestinal features

Participants F, M, S, T, U had presumed diagnoses of abdominal migraines, characterized by stomach pain, nausea, and vomiting. In F, the abdominal migraines were accompanied by cyclic vomiting syndrome. Reports described her episode as significant pain causing writhing with soft, nontender abdomen normal bowel sounds on examination.

#### Endocrinology, metabolism, and immune system function

Short stature is a common phenotype in those with KBG syndrome with up to 66% below the 10th centile in height [5]. Individuals H, J, and O were administered growth hormone. J was born with a length below 1st centile and weight at 57th centile. After receiving somatropin injections from 3.5 years to 5.7 years of age, his height is at the 13th centile and weight is at 24th centile. O was given growth hormone from approximately 6 years to 11 with positive improvement in weight (11th percentile at birth and is now at 45th percentile). Efficacy of hormone supplementation is unknown for H. Reports of precocious puberty, immunodeficiency, recurring infections, allergies are also common.

#### Urogenital features

Urogenital disorders were seen in 48% (*n* = 12) of individuals, with seven being female and five being male. Of note, four males were diagnosed with cryptorchidism. Other diagnoses included abnormalities of the urethra and/or bladder, recurrent urinary tract infection, pollakiuria, polyuria, and enuresis.

#### Dermatologic features

A majority (56%) reported abnormalities of skin, nails, and hair, which included: hirsutism, low anterior hairline or abnormal hair whorl, cellulitis, keratosis pilaris, acne and dry skin, psoriasiform dermatitis, eczema, fingernail dysplasia, and recurrent fungal infections.

## Discussion

We present 25 patients from 22 families with KBG syndrome, molecularly confirmed by identification of variants in *ANKRD11*. Our approach emphasizes data sharing and capitalizes on the increased use and security of videoconferencing technology, allowing access to participants outside of the United States, broadening the generalizability of our results.

Variants in *ANKRD11* are linked to specific facial dysmorphologies; however, the disorder can be difficult to diagnose on facial phenotype alone. While some have a constellation of facial features typical of KBG syndrome, others may look different. This is reinforced by the presence of three different clusters of similar facial characteristics within our cohort detected by GestaltMatcher. There are clear phenotypic overlaps with other genetic syndromes, most notably, Cornelia de Lange Syndrome (CdLS) [24]. CdLS was listed as a differential diagnosis on DeepGestalt for several KBG syndrome photos. The ability of F2G to identify CdLS is 87%, compared with the experts’ average of 77%. When additional photographs were added to the system for increased machine learning, the detection rate of the system increased to 94% [25].

GestaltMatcher identified KBG syndrome as top-10 in 60% of individuals and top-30 for 84% while DeepGestalt identified KBG as top-5 in 80% of cases. The latter “missed” five individuals with molecularly diagnosed KBG syndrome, pointing to the need for greater data collection and training, although as previously noted, three individuals (K, L, and M) are all from the same family, and possess the same missense variant with uncertain pathogenicity.

The PEDIA approach identified KBG syndrome as top-1 rank in 72% of individuals. It outperformed DeepGestalt, which identified 28% with top-1 rank. We envision that an approach integrating facial, feature, and exome analysis could be integrated into future diagnostic pipelines.

We speculate the craniofacial abnormalities and inner ear malformations seen in KBG patients are tied to the high rates of recurrent sinus infection and conductive hearing loss [5], seen in individuals F, H, and U. For example, F and H verbally reported malformed sinus and ear canals, whereas U received a CT of her paranasal sinuses showing right posterior choanal stenosis. However, 32% experienced conductive or sensorineural hearing loss without sinus infection, thus more evidence is necessary to establish correlation. A majority (83%) were diagnosed with chronic otitis media and had concurrent hearing loss— indicating a more likely correlation.

While *ANKRD11* variants have been linked to autistm spectrum disorder [26, 27], none of the interviewed children appeared to have a severe form of autism as they were interactive, social, and maintained eye contact. 28% had been given an ASD diagnosis by previous providers. Quantitative, longitudinal history studies using rating scales are warranted to elucidate how ASD symptoms manifest in KBG syndrome, independent of degree of intellectual disability.

Reduced pain sensation and impaired tactile sensation are previously unrecognized features of KBG syndrome, requiring further investigation. It is not clear whether reduced sensation is due to peripheral or central nervous system impairment. Migraines and abdominal migraines, the latter of which is not a well-known phenomenon, were novel findings in 24% and 20% of our cohort, respectively. The overall prevalence of abdominal migraines in childhood ranges from 2.4 to 4.1% and is more common in females, but the syndrome can be under-recognized in the population [28].

KBG syndrome is associated with cardiac anomalies [5–7] and diagnoses have been made on their detection. Individual B had a persistent left superior vena cava detected at 20 weeks on fetal ultrasound, which prompted karyotyping. Unfortunately, no further genetic work-up was done until five years of age when a KBG diagnosis was made by exome sequencing. Antenatal ultrasound may play a safe and non-invasive role in the detection of KBG syndrome and improve prenatal diagnosis and counseling.

We speculate age of onset of seizures may be inversely linked to severity of developmental delay (Supplementary Text). Systematic and thorough descriptions and reporting of EEG abnormalities can guide physicians in prompt diagnosis. Obtaining a baseline EEG is likely warranted since our data seem to suggest the possibility that the trajectory of those who have seizures compared to those who do not seems to differ, with the latter showing better outcomes. While an ambulatory EEG for 24–72 h is ideal, a routine EEG for 30 min to 1 h could suffice. Future work should include a natural history study assessing age of onset and future levels of overall functioning, and convening an international summit of experts to develop consensus structured treatment guidelines for KBG syndrome.

Overall, the extent and variety of reported deficiencies in KBG syndrome patients could be attributed to the role of *ANKRD11* as a chromatin regulator. Since *ANKRD11* interacts with several key proteins of chromatin remodeling complexes, such as histone deacetylases and acetyltransferases, nuclear co-receptors, etc. and regulates global gene expression [29], it is unsurprising that mouse *ANKRD11* regulates development and/or functioning of multiple tissues [29**–**31], and KBG syndrome patients report systemic phenotypes affecting multiple organs. It is unknown why different KBG syndrome patients tend to have variable number and severity of phenotypes and co-morbidities, although this is likely modified by different genetic backgrounds, environments, and some level of stochasticity.

Some limitations for the present study include the barriers that exist for those who are not familiar with videoconferencing technology. Participants were recruited primarily from referrals from a KBG Foundation Facebook group, further limiting participation to those adept in technology. Examination of stature and teeth morphology was limited over videoconference.

## Conclusions and treatment recommendations

- Early intervention with physical, occupational, and speech therapies is recommended. Anecdotal reports from families indicate that a frequency of at least once weekly is likely ideal. Children with *ANKRD11* variants should undergo baseline auditory screening to rule out hearing defects that might impede speech development.
- There is evidence for the utility of growth hormone treatment for those with short stature (under their target range). Systematic study is required for formal guidelines and recommendations.

- High rates of seizures point at the possible utility of EEG screening upon diagnosis with regular monitoring by a neurologist. Further research is warranted to justify EEG screening, though other rare diseases with a high prevalence of seizures do have formal recommendations for baseline EEG screening, such as Tuberous Sclerosis [32].
- Patients may benefit from cardiac screening (including echocardiography) upon diagnosis.
- Chronic otitis media with hearing loss is a frequent finding. More research is needed to investigate whether aggressive antibiotic treatment could prevent hearing loss.
- Future research and clinical efforts should include more study of GI symptoms (e.g. abdominal migraines) due to their increased prevalence.
- Artificial intelligence-assisted facial applications can play a role in reducing missed diagnoses, given the often mild cognitive deficits and subtle dysmorphic features of KBG syndrome. Combining data from AI and patient registries can optimize diagnosis and help develop guidelines and treatment recommendations.

## Supplementary information

Supplementary Information

Supplementary Table 1

Supplementary Table 2

Supplementary Table 3_

## Data Availability

Data generated and analyzed during this study can be found within the published article and its supplementary files. Additional data are available from the corresponding author on reasonable request. The exome sequencing data were generated as part of clinical testing, so the underlying raw data are not consented for deposition to a public database.
